# Heat in the transport sector: measured heat exposure and interventions to address heat-related health impacts in the minibus taxi industry in South Africa

**DOI:** 10.1007/s00484-025-02935-2

**Published:** 2025-05-13

**Authors:** Caradee Y. Wright, Thandi Kapwata, Siyathemba Kunene, Ngwako Kwatala, Nomfundo Mahlangeni, Tracey Laban, Candice Webster

**Affiliations:** 1 Climate Change and Health Research Programme, Environment and Health Research Unit, South African Medical Research Council, 1 Soutpansberg Road, Pretoria, 0001 South Africa; 2 Department of Geography, Geoinformatics and Meteorology, University of Pretoria, Pretoria, South Africa; 3 Climate Change and Health Research Programme, Environment and Health Research Unit, South African Medical Research Council, Johannesburg, South Africa; 4 Department of Environmental Health, University of Johannesburg, Johannesburg, South Africa; 5 Climate Change and Health Research Programme, Environment and Health Research Unit, South African Medical Research Council, Cape Town, South Africa

**Keywords:** Climate change, Environmental health, Interventions, Low- and middle-income countries, Public health, Temperature

## Abstract

High temperatures, heat and heatwaves are being experienced more frequently and with greater intensity in many parts of the world, including South Africa, and record-breaking maximum temperatures are becoming more common. Exposure to heat has adverse impacts on human health and wellbeing. The transport sector and its users are vulnerable to heat both inside vehicles as well as in places where people wait for public transport. We sought to assess the temperatures experienced in minibus taxis, a common mode of transport in South Africa and in minibus taxi ranks as well as the heat-related perceptions of minibus taxi drivers working in the Chesterville Taxi Association in Durban. We also observed heat-related elements in minibus taxi ranks. Data from temperature loggers showed that temperatures inside minibus taxis reached up to 39 °C and were between 3–4 °C warmer than outdoors. For around 11 h every day, temperatures inside minibus taxis were warmer than 27 °C – the temperature that is linked to heat-health symptoms. Taxi drivers (*N* = 16) all agreed they feel hot in the minibus taxi and more than 90% said they drink water to try to cool down. Taxi ranks were lacking in supply of drinking water and shade for minibus taxis and seating. With the projected increase in temperatures caused by climate change, it is imperative to co-develop mitigation and adaptation strategies to minimise heat-related human health impacts in minibus taxis and taxi ranks especially in low- and middle-income countries.

**Supplementary Information:**

The online version contains supplementary material available at 10.1007/s00484-025-02935-2.

## Introduction

Average global temperatures are projected to rise by 1.5–3 °C by 2050 and by 3–6 °C by 2100 compared to pre-industrial levels (IPCC 2023). High temperatures, such as those occurring on hot days and during heat waves, present a threat to human health and wellbeing and have been associated with several adverse effects (Gasparini et al. 2015; Faurie et al. 2022). These effects include dehydration, heat stroke, respiratory problems, and even impacts on an unborn foetus (such as adverse birth outcomes) (Chersich et al. 2020).

High temperatures pose a threat to vulnerable groups, for example, pregnant women, infants and children, people with pre-existing diseases, the elderly, and outdoor workers. High temperatures are also risky in certain settings, e.g., outdoor work environments. The transport sector is one such setting that is vulnerable to heat—more specifically, the minibus (also known as buses, moto-taxis, matatus, sept-place taxis, tro-tros, keke, danfo, boda boda, etc.) commonly found in low- and middle-income countries (LMICs). This form of public transport is used by large numbers of commuters and passengers with an estimated 80% of the urban population in Africa use some form of public transport (Agyei-Boakye 2024). Despite this being a popular mode of transport, there is a scarcity of research looking at the health repercussions of heat exposure on populations using different modes of transport, especially in LMICs. Some studies have considered urban transport planning in high-income countries (e.g., Nieuwenhuijsen 2020) or analysed air pollution from vehicles (Jones et al. 2016). Paediatric vehicular heat stroke has been studied where children’s thermoregulatory systems are not as efficient as adults and their body temperatures warm at a rate three to five times faster, putting them at risk of overheating when left in a car with the windows sealed (McLaren et al. 2005). On average, 38 children under 15 years of age die from heat stroke after being left in a closed vehicle in the United States each year (Gorucu et al. 2021). Similar information for LMICs is lacking. A recent exploratory review looked at the influence of climate change on the health conditions of land transport drivers, and found impacts associated with cardiovascular and respiratory systems (Hernandez-Duarte et al. 2024). However, it did not consider measurements of heat exposure among the drivers while in the vehicles.

Public transport helps to address global warming by reducing the number of vehicles on the road (Welle et al. 2023) therefore it is important to improve public transport service delivery to meet the needs of the poorest populations who rely on this transport. A recent study spanning Uganda, Zambia, and 15 other African countries highlighted the vital role of climate resilience and inclusive access to transport, emphasising the need for strong political will, cross-sector collaboration, inclusivity for vulnerable groups, and sufficient funding to develop climate-resilient transport infrastructure across Africa (Cinderby et al. 2024). Further research is imperative to understand the health outcomes of heat exposure in transport systems, especially in Africa, to integrate climate resilience and inclusive transport infrastructure that meets the needs of vulnerable populations.

Given the number of drivers, passengers and other key role players in the transport sector, it is important that we begin to understand the possible threats that high temperatures and heat pose to these users. We aimed to answer the following research question: Does heat pose a threat to people in the minibus taxi sector and if so, what can be done about it? There were two research objectives: 1) What are the temperatures inside minibus taxis and do these temperatures pose a potential threat to human health based on published heat-health symptoms at specific temperature thresholds? 2and) Do taxi drivers experience heat and if so, what do they think can be done about it?

To meet these objectives, we measured temperatures inside minibus taxis in the city of Durban, South Africa, and assessed these temperatures in relation to apparent temperature (real-feel temperature that combines temperature and relative humidity) thresholds and potential health threats. We also interviewed taxi drivers to ask about their perceptions of heat and observed heat-related infrastructure (or the lack thereof) in the taxi ranks. Our findings inform recommendations for the transport sector and for those policy- and decision-makers responsible for safeguarding the health and wellbeing of communities. To the best of our knowledge, this is the first study to assess temperatures inside minibus taxis in South Africa and possibly Africa.

## Materials and methods

### Study area

**Fig. 1:**
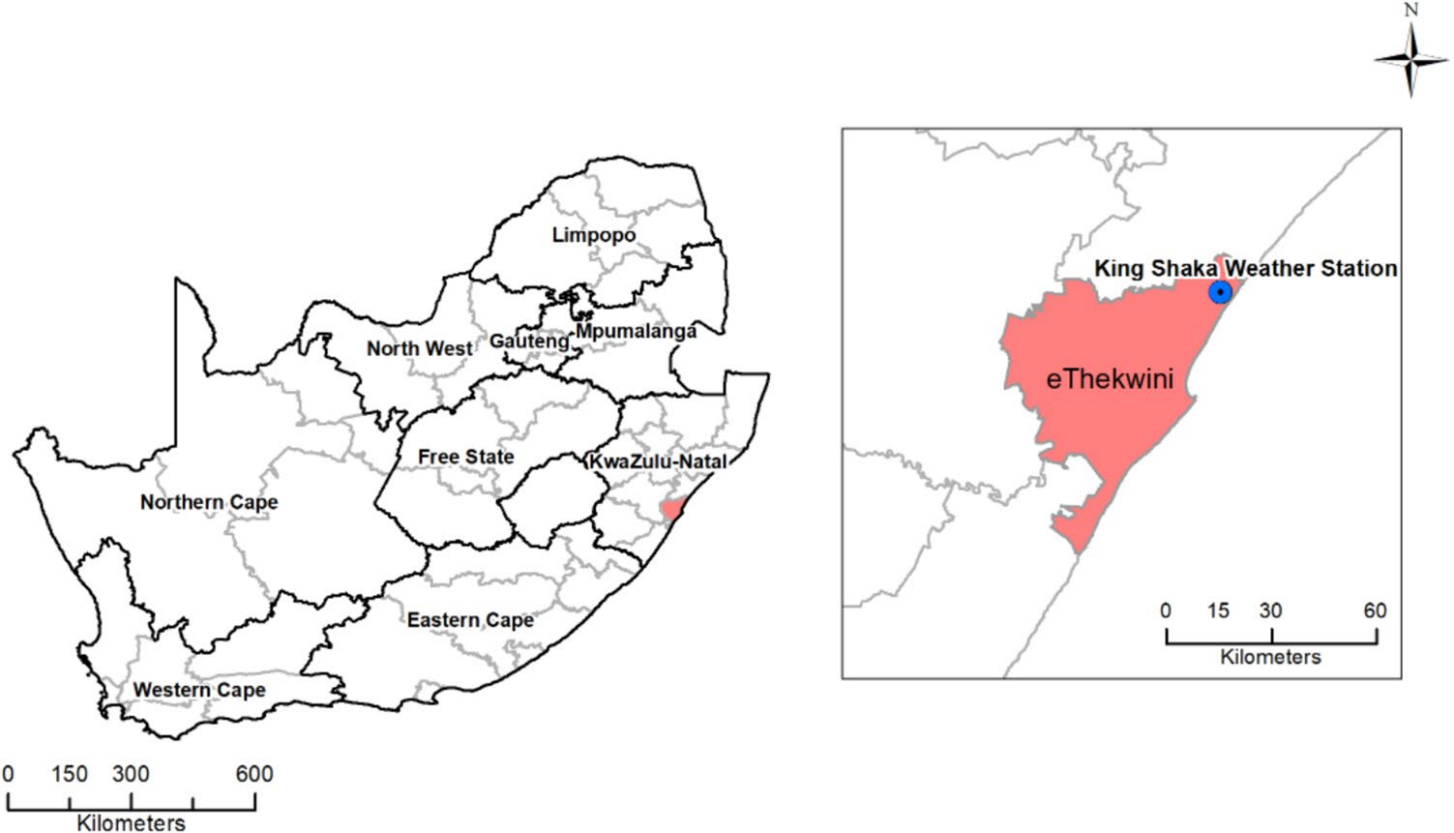
Location of eThekwini Municipality and the SAWS King Shaka Airport Weather Station. The distance between the study sites in eThekwini and the Weather Station is approximately 30 km

The study was conducted in Durban (now known as eThekwini) a metropolitan municipality in the KwaZulu-Natal Province of South Africa (Fig. 1). Durban is the third most populous city in South Africa with a population of 4.2 million people (National Government Handbook – South Africa 2021). The population density is 2 600 people per km^2^ in a total area of 2 556 km^2^.

Table 1 presents the climatic conditions of Durban (Climate Data 2025). The climate in Durban is characterised as a humid subtropical climate with hot humid summers and warm moderate dry winters (Koppen climate classification Cfa) (Kottek et al. 2006). Durban is bordered by the Indian Ocean and inland, and as a result, the differences of summer maximum and winter minimum temperatures are comparably small. Durban lies at an altitude of 101 m above sea level. The influence of the surrounding ocean and topography creates micro- and macro-climates in the area. The average mean temperature ranges between 13 °C and 27 °C (Mncube et al. 2023). Weather systems such as coastal lows and El Nino events tend to increase temperatures. In addition, heatwaves (with a threshold of 34 °C in Durban) (Mbokodo et al. 2020) have been more common in the city than ever before, which might be due to the effect of climate change.

**Table 1:** Climatic conditions in Durban

Metric	Value
Average summer temperature	24 °C
Average winter temperature	17 °C
Winter nighttime temperatures	10 °C
February (hottest month) average temperature	25 °C
June (coldest month) average temperature	18 °C
Annual rainfall	1009 mm
Summer sunshine hours	7–8 h
Winter sunshine hours	6–7 h

### Data collection

This study was based on qualitative and quantitative research methods, namely temperature measurements inside taxis and taxi ranks, questionnaires administered to participants, and surrounding environment observation. Prior to the execution of the fieldwork, the data collection protocols were piloted in the City of Johannesburg among commuters and drivers of minibus taxis. The main study was conducted in Durban from 19 March—20 March 2024 (done as soon as possible after funding was received to try and carry out the work during the early part of autumn when temperatures were likely to still be relatively warm) when temperature and relative humidity levels were measured in 16 minibus taxis and 12 ranks, and a taxi driver questionnaire and taxi rank observation checklist were administered. The taxi driver questionnaire was developed by the research team to gather the experiences of taxi drivers associated with heat during their daily working routine. In-person questionnaires were administered to taxi drivers and took approximately 10 min each. Fieldworkers entered the taxi drivers’ responses on RedCap software installed on the tablet. The taxi rank observation checklist was completed by fieldworkers on RedCap software while on site.

### Sampling procedure

The sampling procedure was done, with permission, from the Chesterville Taxi Association (CTA) taxis operating from Durban central to other destinations. At least one taxi was selected per taxi route in which the CTA operates. Taxi routes were selected to maximise contrasts in heat levels to the different environments. The CTA operates along seven taxi routes around Durban.

The inclusion criteria for installing temperature sensors inside a minibus taxi included: (i) a Toyota HiAce minibus, (ii) nothing should be placed under the seats (e.g. speakers), (iii) availability of empty space for deployment on the third-row seat and last row seat and (iv) the taxi should operate daily. Taxis operating less than four days a week were excluded.

The inclusion criteria for installing a temperature logger at a taxi rank included: (i) it was within ten metres from where the taxis were parked and (ii) there were at least more than three taxis queuing to pick up passengers at any given time.

**Fig. 2:**
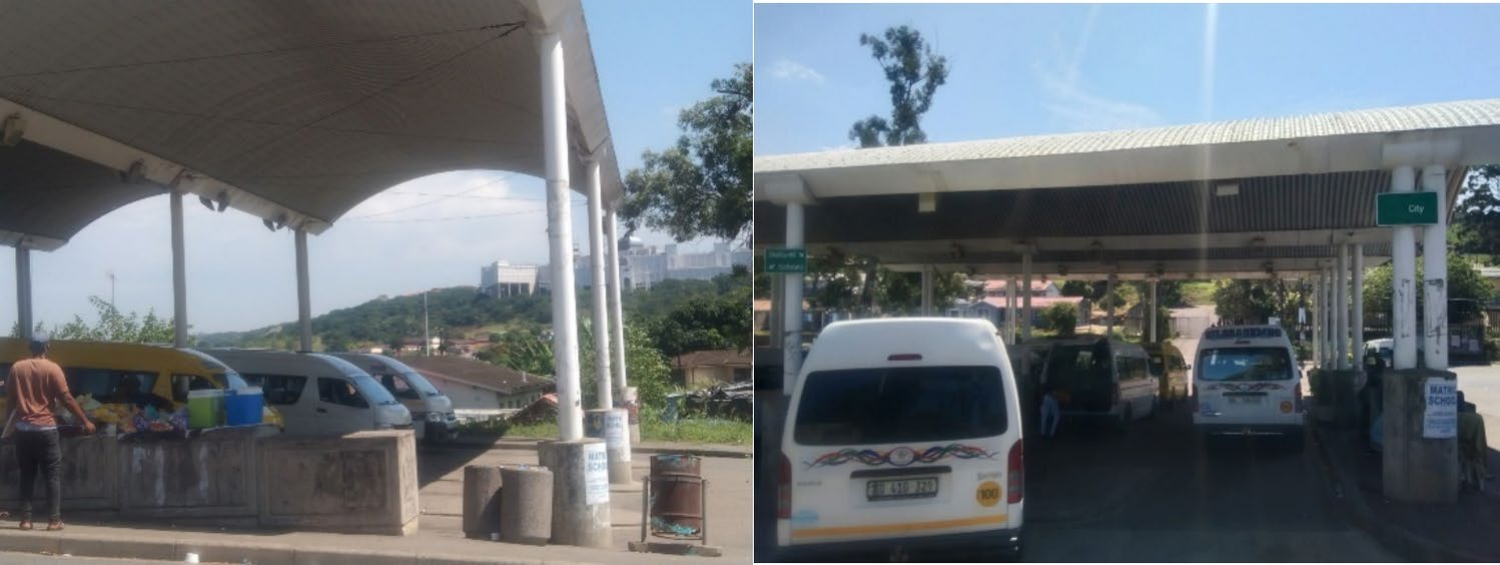
Some of the minibus taxi ranks included in the study

The study was conducted across 12 taxi ranks that service seven taxi routes in Durban. Figure 2 illustrates the nature of the taxi ranks included in the study. Four taxi ranks were classified as main taxi ranks while eight taxi ranks were classified as satellite taxi ranks. Main taxi rank was defined as a piece of land with approved land zoning certificate and sheltered taxi rank structure, while a satellite taxi rank was defined as land with a different zoning but used as an informal taxi rank without infrastructure (i.e. shelter, rest rooms). Of the 12 taxi ranks, four of the taxi ranks were located in the city centre, five taxi ranks were located in the shopping centres and government institutions (i.e. hospital or university), and three taxi ranks were located in the residential areas. Toyota HiAce model was selected to partake in the study as they are a popular model. This study’s sampling size was 16 taxis at most due to the availability of sensors; thus, no randomised or stratified sampling was used.

### Temperature and relative humidity data collection

The Thermochron iButton is a low-cost sensor used to measure temperature and humidity. The device has a temperature resolution of approximately ± 1 °C, and its sensitivity range is −30 °C to + 70 °C for temperature (iButton Link Technologies 2024) – no information was provided by the manufacturer regarding relative humidity. iButtons can store 2 048 temperature data points at a sampling rate of 1 to 255 min. The device is the size of a small disc battery and is powered by a lithium battery that should operate for at least 10 years without maintenance. The device is held in a specially designed plastic fob and a steel chip is inserted on a plastic tag. Temperature and humidity are measured by the steel chip and the device saves the recorded data in-situ. The size of the tag is approximately 8 cm in height and 2 cm width. The tag has a small opening that is used to secure the iButton with a cable tie.

iButtons were installed on 19–20 March in 16 taxis and removed on 22—23 March in taxis, while iButtons were installed on 20 March in three taxi ranks and removed on 23 March from the taxi ranks. The iButtons were programmed to record temperature and relative humidity every hour. To access the data recorded, the iButton was loaded on a software interface to download the data and save the data in a readable format. Each iButton was marked with a unique serial number and was programmed using the software supplied. The device does not have an ON/OFF switch; however, the device was set to start measurements at a specified date and time using the software supplied.

**Fig. 3:**
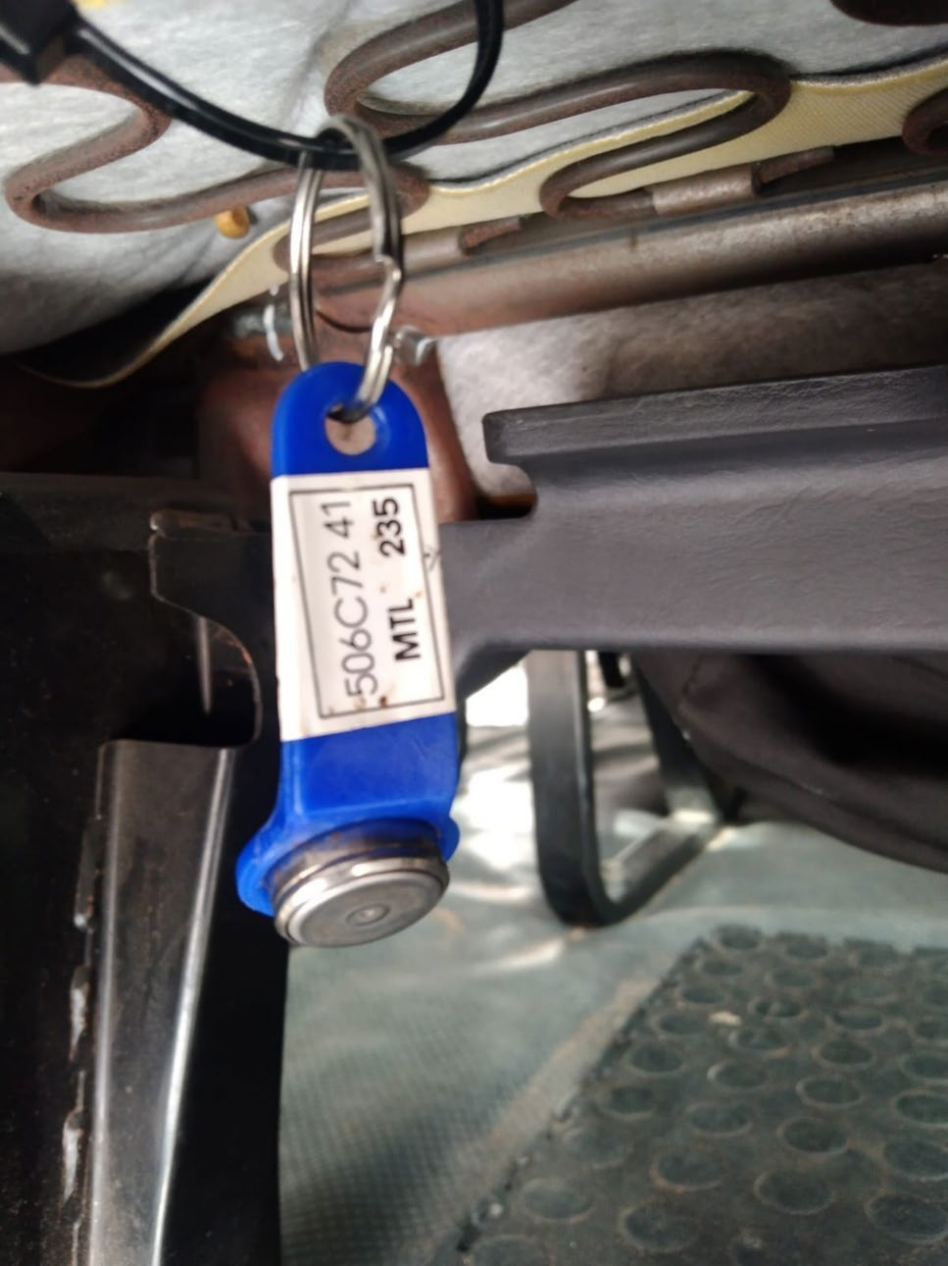
The location of the temperature logger under the seat in the minibus taxi

For this study, iButtons were installed in different settings. The main placement for measurements was inside the taxi (Fig. 3). However, we also installed iButtons outdoors in taxi ranks but these iButtons did not record correctly and only one set of data from one rank was included in the study – this logger was placed in the shade at the taxi rank. Cable ties were used to secure the iButtons in place and ensure they could not be moved around or fall off. The taxi drivers were requested to check for the sensor daily and report if devices were missing. The date and time of installation of the iButton were recorded on log sheets for record keeping purposes. Data quality control procedures included checking for incorrect/impossible readings, completeness of data and deleting data recorded before the installation date and time as per the log sheets records.

Data were presented descriptively and a Pearson correlation analysis was conducted to investigate the relationship between temperatures inside taxis and outdoor temperature measured in the taxi rank where p < 0.05 was considered statistically significant.

### Apparent temperature calculations

We used temperature and relative humidity measurements to calculate apparent temperature. While other indices now exist (Streinu-Cercel et al. 2008; Pantavou et al. 2024), apparent temperature is an indicator that is also used to measure thermal comfort (Steadman 1979). It combines temperature, wind speed and humidity to obtain a metric that is considered as “real-feel” i.e., what the temperature feels like to the human body. The equation is as follows:

$$AT=Ta+0.33*e-0.70*\text{ws}-4.00$$

where:

Ta: dry bulb temperature (°C).
e: water vapour pressure (hPa).
ws: wind speed (m/s) at an elevation of 10 m (set to 0 for an indoor setting).

and:

$$e=rh/100*6.105 * {e}^{(17.27*Ta/(237.7+Ta))}$$

where rh = relative humidity (%).

Apparent temperature can be interpreted according to the four classifications in Table 2 (Steadman 1979). Possible health effects or symptoms for apparent temperature were evaluated using temperature ranges developed by the United States National Weather Service (USNWS) and the National Oceanic and Atmospheric Administration (NOAA) (Steadman 1979). These ranges are categorised into ‘caution’, ‘extreme’, ‘danger’, and ‘extreme danger’ and potential health effects, described as symptoms and endopints, are also provided for each warning level.

**Table 2:** Apparent temperature thresholds and potential health impacts (after Steadman 1979)

Symptom Band	Classification Apparent	Temperature Range (°C)	Possible health effects on the body
I	Caution	27–32	Fatigue possible with prolonged exposure and/or physical activity
II	Extreme caution	33–39	Heat stroke, heat cramps, or heat exhaustion possible with prolonged exposure and/or physical activity
III	Danger	39–51	Heat cramps or heat exhaustion likely, and heat stroke possible with prolonged
IV	Extreme danger	> 51	Heat stroke highly likely

### Heat metrics and calculations

Exposure metrics are quantitative values that indicate an individual’s exposure to environmental heat. Two individual experienced temperatures (IET), namely, Maximum IET and Longest Exposure Period (LEP) were applied to estimate an individual's heat exposure (Hondula et al. 2021). The intensity of heat exposure is best determined by Maximum IET while the duration of heat exposure is best determined by LEP.

Maximum IET refers to the hottest hour in a given time:

$$\text{Maximum IET}=\text{maximum value of temperature observations}$$

where:

Maximum value of temperature observations is the highest observed temperature reading from the observations. Maximum IET is effectively the maximum of an individual participant.

LEP refers to the continuous amount of time the individual is exposed to heat above the temperature threshold. LEP is determined by calculating the continuous hours above the temperature threshold; and ranking those hours by ascending order. The highest number in the ascending order is LEP.

The two IET exposure metrics were compiled using mean of means which translates to finding the mean of the observations for each participant and deriving the metrics from the means instead of individual observations. This study had a sample size of 30 (two loggers per minibus taxi). The time frame (day hours) for hot days were used in the study. Day hours refer to the moment of sunrise to sunset (i.e., 6am – 6 pm in summer months). There were four observed hot days during the study (e.g. 19, 20, 22, 23 March 2024). The 21 March 2024 was not regarded as a hot day due to low temperatures with the minimum and maximum temperatures of 21 °C and 24 °C, respectively.

### Questionnaires for heat perceptions and interventions

The taxi driver questionnaire was conducted using RedCap software on 19 March—20 March 2024. The questionnaire was developed by the research team to understand the experiences of taxi drivers associated with heat during their daily working routine. In-person questionnaires were administered to drivers and took about 10 min to complete.

The questionnaire is included in the supplementary material. It was grouped into four themes comprising questions concerned with (1) demographics, (2) personal experiences with heat, (3) thoughts on the rank structure and lack of, and (4) ways in which heat can be mitigated in the taxi and the rank. The demographics information collected was age and gender; individual heat experience while inside the taxi or at a taxi rank, times of day when heat is high, heat coping mechanisms used to alleviate body heat and heat inside the taxi, and advice on actions to keep the body and the taxi cooler; thoughts on rank structure and taxi design information collected was an open-ended question where taxi drivers share their knowledge and thoughts on improving the cooling at taxi ranks and in taxis. Fieldworkers entered the participant responses using RedCap software installed on a tablet.

### Taxi rank observation checklist

The taxi rank observation checklist (also included in the supplementary material) was conducted from 19 to 20 March 2024 by the fieldworker. The taxi rank observation checklist was used to capture the surrounding environment and attributes of a taxi rank. The observation sheet was divided into three themes: (1) taxi rank infrastructure, (2) commuter needs and accommodation, and (3) land cover. Data collected on the taxi rank infrastructure included the type of taxi rank, peak service times, taxi rank shelter, and number of taxis operating in the taxi rank. Data collected on taxi rank commuters included responses to questions pertaining to the presence of shade in the waiting area, seating area, vendor stalls, toilet facilities, and running water. Data collected on taxi rank land use included the number of trees around the taxi rank, the type of vegetation around the taxi rank, and the type of land cover.

### Research ethics approval

Since this study entailed working with participants, the study protocol was reviewed and approved by the South African Medical Research Council Human Research Ethics Review Board (Ethics number: EC019-9/2022).

## Results

### Temperatures measured inside minibus taxis

Temperatures inside taxis during the study period ranged from 19 °C – 39 °C with a mean of 27 °C. The lowest temperatures were observed between the 21 and 22 of March. Average hourly temperatures inside minibus taxis displayed a cyclical pattern with low temperatures observed in the early morning and evening and peaks occurring between 12:00–15:00 in all taxis.

### Apparent temperatures and possible health effects or symptoms when inside minibus taxis

**Fig. 4:**
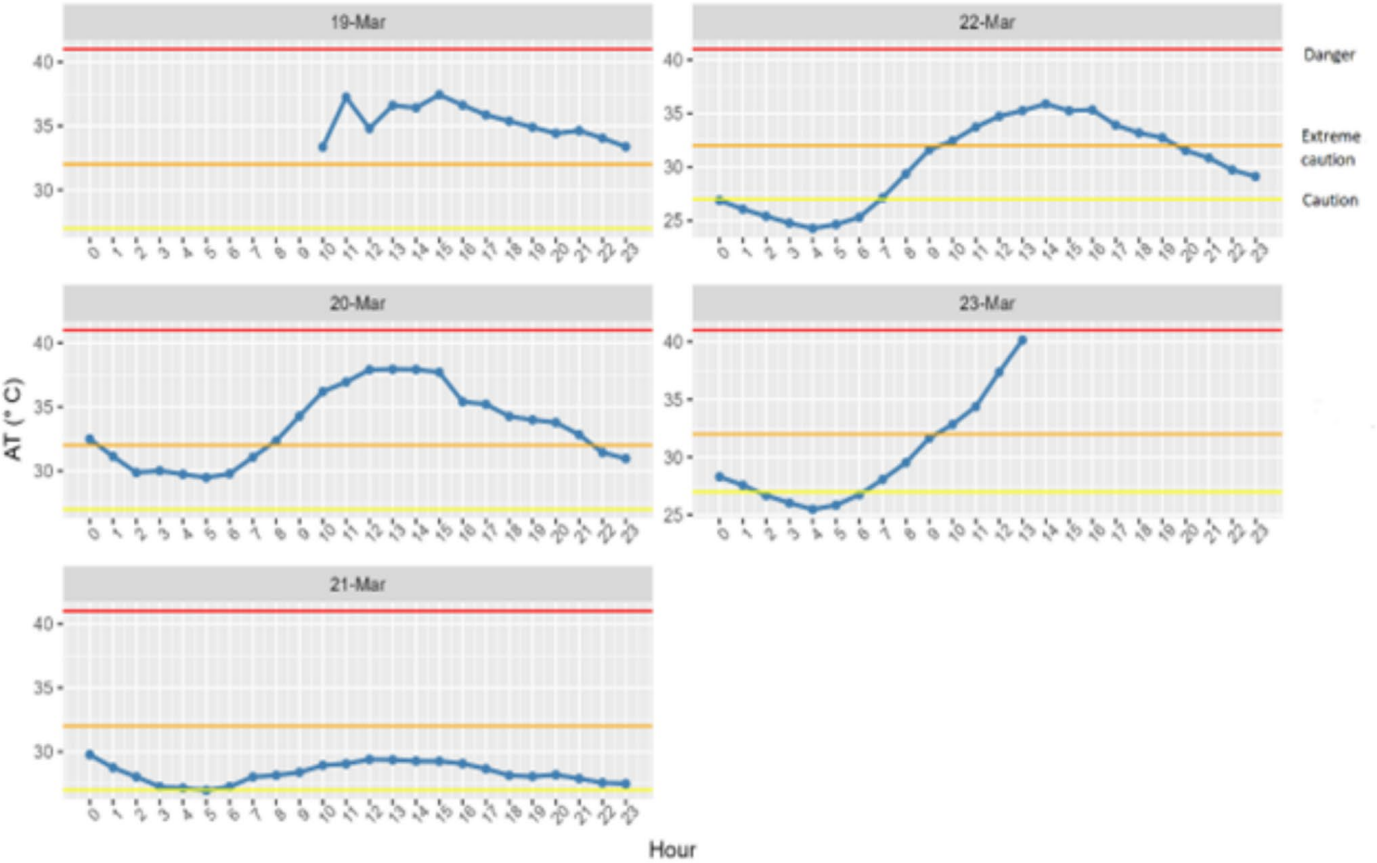
Mean apparent temperatures (AT) (blue line) measured in the minibus taxis for each day of the study. The red line indicates danger, orange line is extreme caution and yellow line is caution in relation to the heat-health thresholds

Daily mean apparent temperature inside taxis was above 27 °C on all five days, with the highest mean being 35 °C on the 19 March (Fig. 4). Therefore, taxi drivers were exposed to mean apparent temperatures that could result in health impacts ranging from mild (fatigue and discomfort) to moderate/severe (heat stroke, sun stroke, heat cramps, or heat exhaustion) (see Table 2). The plots for 19 and 23 March are incomplete because these were the iButton installation and removal dates. While we tried to conduct our fieldwork during warm weather, temperatures did begin to cool towards the end of the data collection period, as seen on the 21 March. This is typical of the period when the seasons are changing from summer to autumn in Durban.

### Apparent temperatures measured in taxis compared to temperatures in a taxi rank

**Fig. 5:**
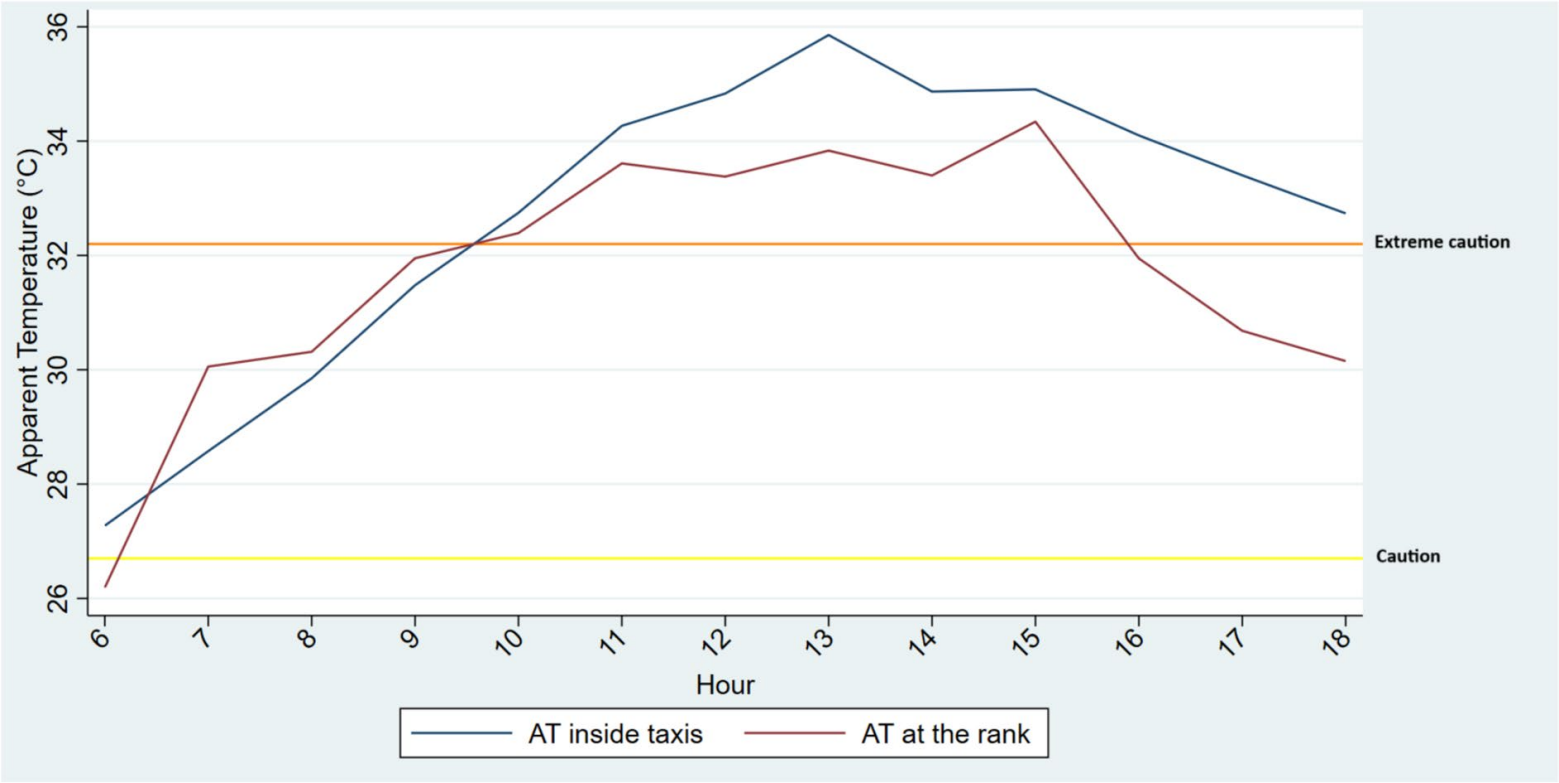
Combined average hourly apparent temperature for inside minibus taxis and at the taxi rank during the study period

Figure 5 shows the apparent temperature inside taxis and at the taxi rank where the taxis were parked. Apparent temperatures were lower in taxi ranks compared to inside taxis which suggests passengers were possibly at reduced threat of heat-related symptoms while waiting outside (so long as they were not standing in direct sunlight) for a taxi compared to when they were inside the taxi. Outdoor temperatures were generally below all apparent temperature symptom thresholds (see Table 2). However, apparent temperatures inside taxis were within the “caution” and extreme caution” bands.

### Maximum IET and LEP estimations for individual heat exposure

The statistics summary was compiled for maximum IET and LEP using four hot days (excluding 21 March which was colder) from 6 h00 to 18 h00 (Table 3). We calculated the mean for each iButton in every taxi; and from the calculated means we extracted the descriptive statistics. The maximum of mean IET was 32.5 °C. Maximum max IET was 43.6°. LEP is the maximum continuous duration that the taxis are exposed to heat or the maximum cumulative hours of exposure above threshold. The descriptive statistics, particularly the mean LEP and max LEP over temperature threshold of 30.6 °C were 5 h and 10 h per four days, respectively.

**Table 3:** Summary statistics of heat exposure metrics from four days inside taxis in Durban

Metric	Mean	Min	Max	Median	Std dev
Max IET (°C)	34.4	23.6	43.6	34.6	3.5
LEP (minutes)	5.3	0	10.0	6.0	2.7

### Questionnaire findings

The taxi driver questionnaire sought to understand the demographics and heat experiences of taxi drivers (Table 4). Taxi driver questionnaires were administered to 16 taxi drivers and they typically reported feeling hot (sometimes or always) in the minibus taxis. All taxi drivers were men and the majority (56%) were younger than 45 years. At least 69% of taxi drivers worked more than five hours daily. All taxi drivers responded that they experienced heat in their line of work and most (93%) taxi drivers either open a taxi window or drink water in an attempt to cool their bodies down.

**Table 4:** Results of the questionnaire survey among the minibus taxi drivers

Total number of taxi drivers (*N* = 16)	Frequency	
Question	Number (n)	Percentage (%)
How old are you?		
18–25 years	0	0
26–35 years	9	56
36–45 years	5	31
46–55 years	1	6
56–65 years	0	0
Older than 66 years	0	0
When you are driving or travelling in the taxi, do you feel hot?		
Never	0	0
Sometimes	10	63
Always	6	37
When you feel hot, do you do something to feel cooler?		
Yes	15	94
No	1	6
Do you use the fan setting in the taxi?		
Never	13	81
Sometimes	2	13
Always	1	6
Do you feel it is hotter inside the taxi rank or outside?		
Inside	9	56
Outside	7	44
What are the pick times at the rank?		
1–4 h	1	6
5–8 h	6	38
9–12 h	5	31
13–16 h	4	25
What do you do to cool down when it’s hot in a minibus taxi? You can mention as many ways of coping as you like		
Drink water	15	94
Drink cold drinks/fizzy drinks	4	25
Drink an energy drink	4	25
Take off an item of clothing	5	31
Fan yourself (e.g. with your hand, a paper)	2	12
Open a window	15	93
None of the above	1	6
Other (discussed in text) *i.e. place a towel over their heads*	2	12
How many hours a day do you spend inside a taxi rank?		
1–4 h	5	31
5–8 h	6	38
9–12 h	4	25
Missing data	1	6

Taxi drivers also provided suggestions on what they think could be done to help reduce heat inside taxis and at taxi ranks. Their suggestions included putting air-conditioning inside the minibus taxis, have more shelters (not metal which traps heat) for undercover parking, bigger ranks to allow for more air circulation, and more trees. Other suggestions were to restrict areas for minibus taxis and vendors in ranks to accommodate for sufficient ventilation as well as provide a consistent supply of drinking water from taps in rest rooms.

### Taxi rank observations in terms of heat protection

Seventy percent of the taxi ranks were covered with high heat-absorbing surfaces (i.e. tar, concrete, and paving). Only 25% percent of taxi ranks had shelters. Custom designated shelter roofs differed by pattern and height. Corrugated iron was the most common roofing material used, followed by concrete for the taxi rank located at the ground floor. Custom designated shelters had queueing rails for commuters, vendor stalls, restroom facilities, and rubbish bins, however, no running water.

Seventy five percent of taxi rank did not have a rank shelter. Taxis waited in the sun or under tree shade if the direction of the sun allowed. Informal taxi ranks had no reticulation therefore no running water was available. Some taxi ranks with no custom rank shelter had a commuter waiting shelter, while others had trees for shade, and others had no shade at all. Ninety one percent of taxi ranks with no custom rank shelter had enough space to install the rank shelters in the future.

## Discussion

The impacts of heat on health and wellbeing extend from worsening physiological outcomes to affecting mental health (Ebi et al. 2021). Vulnerability to heat is influenced by factors such as age, health status, occupation and socio-economic conditions (Jurgilevich et al. 2023). Consecutive days of cumulative heat exposure increase physiological (i.e., increased core temperature, heart rate, etc.) and perceptual (i.e., perceived exertion, thermal discomfort, etc.) heat strain (Schlader et al. 2017). Cumulative heat exposure above specific thresholds accelerates a decline in cognitive performance quicker in African-male populations and people who reside in disadvantaged neighbourhoods (Choi et al. 2023). The taxi industry is modelled in a way that minibus taxi drivers must work long hours, reducing their chances of visiting health facilities leading to possible delayed diagnosis and treatment for physical or cognitive disorders potentially associated with heat exposure. Cognitive disorders or reduced cognitive performance increases the potential risk of road accidents and body injuries (Schlader et al. 2017). Cumulative heat exposure above heat-specific threshold temperatures for consecutive days may also lead to thermoregulatory impairments due to a decrease in thermoregulatory functions (Notley et al. 2018).

Our study found that taxi drivers may have experienced dehydration, heat-related illnesses or exacerbation of existing chronic conditions because of the high apparent temperatures they were exposed to and the long duration of exposure, however, we cannot be sure since we did not do physical health assessments. Temperatures inside taxis reached 39 °C and mean relative humidity in taxis during the study period was 72%. These values exceed the thresholds of 32 °C for temperature and 50% for humidity which were found to be associated with extreme to lethal heat stress during extended exposure (Asseng et al. 2021). Previous studies show that the impacts of heat last for as long as four days post-exposure (WHO 2024) further compounding the possible health threats faced by taxi drivers.

Temperatures inside taxis were consistently higher than outdoor temperatures. This highlights the need for attention to be directed towards the design and implementation of heat reduction or prevention strategies inside public modes of transport. This is particularly important in the context of climate change as rising outdoor temperatures are likely to further increase temperatures inside taxis.

Driving is an occupation that requires immense concentration and the minibus taxi drivers in the taxis that were part of our study were exposed to apparent temperatures (real-feel temperatures combining temperature and relative humidity) between 24–40 °C. Heat-health guidelines for workplaces state that once apparent temperature reaches and exceeds 40 °C, dehydration and possible heat exhaustion are significantly increased, resulting in a reduction in productivity and increased errors (WHO 2024). Therefore, both passive and active cooling interventions should be considered to increase thermal comfort inside taxis. Active cooling interventions include fans and air-conditioning functions in the minibus taxis while passive cooling options are shelters and awnings made from non-metal products and making taxi ranks larger to allow for better ventilation.

Support for heat-protective spaces such as minibus taxi ranks in cities is needed and a multi-sector, multi-dimensional approach is likely to be appropriate. There are roles for the minibus taxi associations, the city officials in transport, environment and climate change, the minibus taxi manufacturers together with the minibus drivers themselves. With adequate support and funding, several recommendations are proposed for these sectors to prevent heat-related health threats in this sector. For example, in the HiAce minibus vehicle, the positioning of the engine under the driver could be reconsidered to reduce heat from the engine contributing to hot temperatures inside the taxi. The windows could be tinted to reduce sunlight from entering the interior of the taxi and warming temperatures inside the taxi. Fans could be placed at the front, middle and rear of the taxi to ensure air movement and windows should open wide to allow ventilation.

In the taxi rank, there should be adequate water for consumption readily available at drinking fountains or, at the very least, bathrooms with taps from which people can drink water. The city could also provide cooling centres where taxi drivers and communities can sit or stand to wait for the next trip. A cooling centre usually has fans or air conditioning.

### Study limitations and research recommendations

While our study had strengths, such as gathering data in an overlooked transport sector, there were some limitations. Data loggers are low-cost sensors and may be unreliable. Data from inside taxi ranks were lost due to loggers malfunctioning. In the future, two loggers should be installed at the same place to ensure backup data are available. We applied apparent temperature, max IET and LEP. Apparent temperature was selected since our loggers measured temperature and relative humidity. Apparent temperature may not be adequate to replicate ‘real-feel’ human thermal sensation since it does not consider metabolic rate, clothing insulation, radiative heat exchange of human body, etc. Other studies may consider other thermal comfort indices like physical equivalent temperature (PET), universal thermal climate index (UTCI) and even mean radiant temperature as an indicator of human heat stress.

There are several possibilities for further research. Minibus taxis exist across the country (and continent) and this means an opportunity for a bigger study to be conducted simultaneously across the country during the summer to increase the sample size. Also, many people travel by minibus taxi from urban homes to rural homes leading to very full minibus taxis and congestion. Data from a study like this could be used to link health assessments with heat exposure to show the link between heat and health. To address the methodology used, future studies can place loggers in various points around the inside of the minibus taxis. Participants’ recommendations could be tested by introducing minibus taxis with air conditioning and windows that open fully.

Studying different taxis rank structures, formal and informal, to assess the difference in temperatures and to consider using multiple participants such as taxi rank marshals to get different perspectives should also be considered. There is the potential of linking the loggers’ data to the SAWS weather station or installing mobile weather stations at taxi ranks to check the difference between the outside and inside temperatures, however, challenges such as taxis not being centrally located all the time and the safety risk of placing mobile weather stations at taxi ranks will have to be addressed. Awareness can be raised through stakeholder engagement and distribution of awareness materials (e.g., flyers, posters). Examples of these were prepared for the City of Tshwane (Pretoria) and are included in the supplementary material.

## Conclusions

The aim of this study was to understand heat exposure among drivers in the minibus taxis in the city of Durban to recommend context specific adaptation actions to reduce adverse impacts on taxi drivers and passengers. This study found that taxi drivers experience temperatures above 30 °C and such heat exposure could impact their health during taxi journeys or while waiting for passengers. Additional research to conduct physical health assessments would help better understand potential health impacts for the drivers. Currently, their heat coping mechanisms include opening a taxi window, or drinking water to cool down and stay hydrated. However, the lack of access to water, either water foundations or washrooms, at taxi ranks is a significant concern as remaining hydrated is crucial to preventing heat stress.

The current taxi rank infrastructure, such as shelters overhead the taxis and in some instances trees, is not able to accommodate the high volumes of taxis and passengers and provide the necessary shade. Taxi ranks are often built without consideration of shade. With more than 16 million people in South Africa making use of minibus taxis every day, including drivers, marshals and commuters, the potential burden of heat-health related impacts is great. With the projected increase in temperatures associated with climate change, it is imperative to co-develop mitigation and adaptation strategies to minimise heat-related human health effects in minibus taxis and taxi ranks. This requires an integrated, participatory, and context-specific approach. All stakeholders including taxi drivers and associations, commuters (especially the elderly, children and pregnant women), local governments including transport, health and climate, urban planners, researchers and non-governmental organizations, should be engaged as early as possible in the planning processes and design of taxi ranks. Heat mapping should be conducted to assess specific vulnerabilities and surveys of drivers and commuters should entail gather information about heat exposure symptoms, daily routines and peak heat times and cooling practices. High-risk groups should be identified, such as long-distance drivers and commuters in poorly ventilated taxis.

**Fig. 6:**
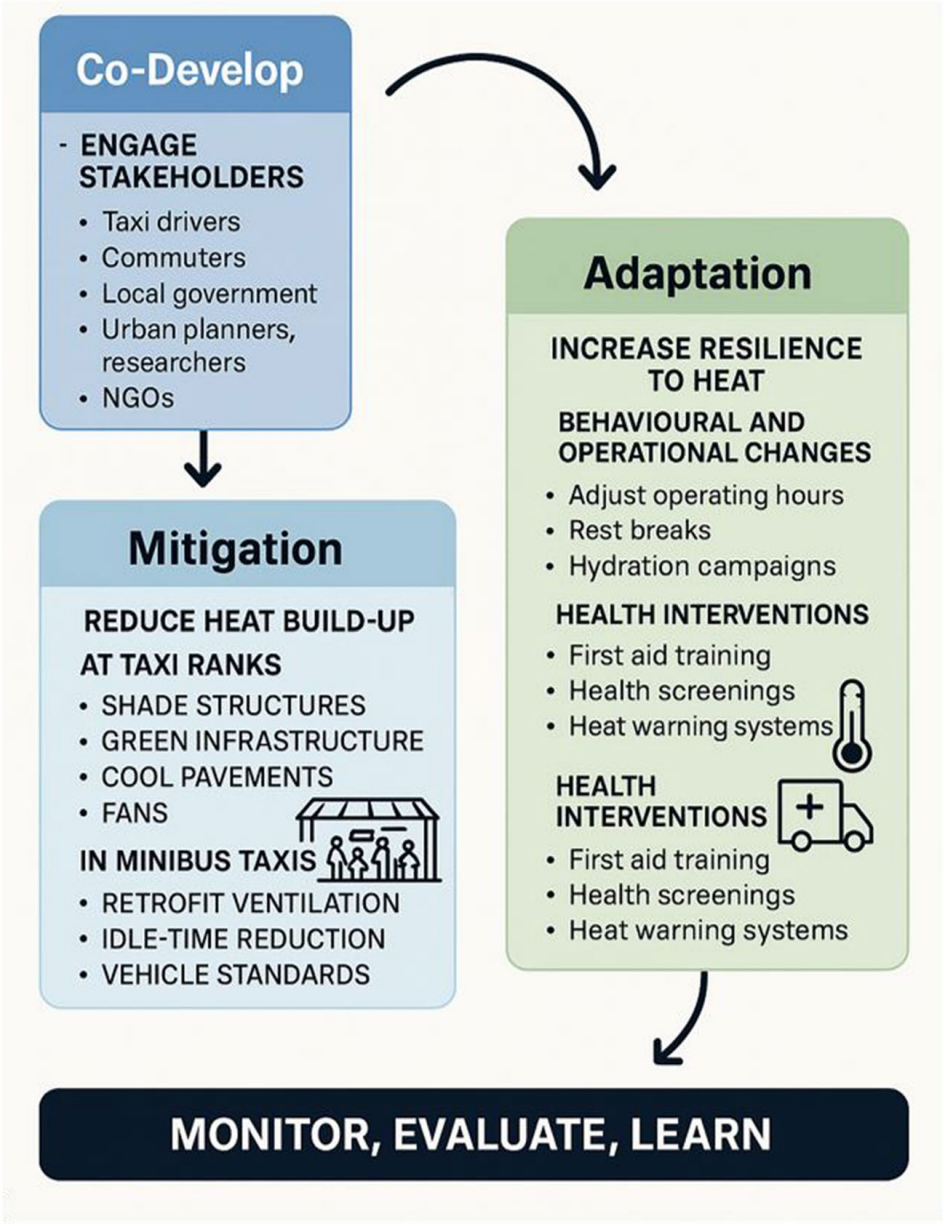
Co-developing mitigation and adaptation strategies to minimise heat-related health risks in minibus taxis and taxi ranks

Mitigation and adaptation strategies to reduce heat build-up and increase resilience to heat, respectively, must also be considered (Fig. 6). At taxi ranks, installing canopies with heat-reflective materials, trees, green roofs or vertical garden walls would help reduce heat. In minibus taxis, the installation of roof vents, window tinting and reflective insulation will help to reduce heat exposure. Drivers should be encouraged to turn off engines during long waits and reduce idle time. Several adaptation strategies are also possible including rest breaks for drivers during high heat periods, and hydration campaigns that provide free water and education on heat stress symptoms. Health interventions that may help are equipping drivers with first aid training to identify heat stress, providing regular health screenings to check for heat-related symptoms and implementing a heat warning system tied to local weather data. For these interventions to be sustained and successful, co-development is essential using activities such as community workshops, mobile surveys and design sprints to prototype shade and rank design with users.

## Supplementary Information

Below is the link to the electronic supplementary material.

Supplementary file1 (PDF 38.8 KB)

Supplementary file2 (PDF 39.2 KB)

Supplementary file3 (PDF 44.1 KB)

Supplementary file4 (DOCX 19.9 KB)

Supplementary file5 (PDF 480 KB)

Supplementary file6 (PDF 0.99 MB)

Supplementary file7 (PDF 138 KB)

Supplementary file8 (PDF 926 KB)

## Data Availability

The datasets generate during and/or analysed during the current study are available from the corresponding author on reasonable request.
