# Supplementary material for: Heat in the transport sector: measured heat exposure and interventions to address heat-related health impacts in the minibus taxi industry in South Africa
Source: Int J Biometeorol. 2025 May 13;69(10):2475–87. doi: 10.1007/s00484-025-02935-2 (PMC12540607; doi:10.1007/s00484-025-02935-2)
Supplement: Supplementary file 1 — Supplementary file1 (PDF 38.8 KB) [file 484_2025_2935_MOESM1_ESM.pdf]

Taxi Driver Questionnaire Durban Be2f02

Record ID	______________________
Unique Questionnaire Identity Number	______________________
Are you:	◯ Male<br>◯ Female<br>◯ Other
How old are you?	______________________
Have you ever been told that you have:	◯ Heart attack or coronary heart disease<br>◯ Rheumatic heart disease or other heart problem<br>◯ Stroke<br>◯ Cancer<br>◯ High blood cholesterol<br>◯ Diabetes/elevated blood sugar<br>◯ High blood pressure<br>◯ Kidney failure<br>◯ Mental health problems like depression,anxiety, stress<br>◯ None of the above
How many hours a day do you spend inside a taxi?	______________________
When you are driving or travelling in the taxi, do you feel hot:	◯ Never<br>◯ Sometimes<br>◯ Always
Have you ever experienced any of the following symptoms when you feel hot while driving/sitting in the taxi?	☐ Sweating<br>☐ Heat cramps<br>☐ Headaches<br>☐ A faster heartbeat<br>☐ Nausea / vomiting<br>☐ Heat rash / pimples or blisters on skin<br>☐ Heat exhaustion / feeling tired or weak<br>☐ Dizziness or confusion<br>☐ Difficulties breathing<br>☐ Fainting<br>☐ Irritability<br>☐ None of the above<br>☐ Other - open-ended
Are these symptoms worse in the morning or in the afternoon?	☐ Morning<br>☐ Afternoon
When you feel hot, do you do something to try and feel cooler?	◯ Yes<br>◯ No

What do you do to try and feel cooler when you feel hot in the taxi? You can mention as many ways of coping as you like.	☐ Drink water<br>☐ Drink cold drink / fizzy drinks<br>☐ Drink an energy drink<br>☐ Take off an item of clothing<br>☐ Fan yourself (eg with your hand, a paper)<br>☐ Open a window<br>☐ None of the above<br>☐ Other: open-ended
Other - Heat coping mechanism	__________________________
Do you use the air conditioner in the taxi:	◯ Never<br>◯ Sometimes<br>◯ Always
Do you use the fan setting in the taxi:	◯ Never<br>◯ Sometimes<br>◯ Always
How many hours a day do you spend inside the taxi rank?	__________________________
Do you feel it is more hot inside the taxi rank or outside?	◯ Inside<br>◯ Outside
Have you ever experienced any of the following symptoms when you feel hot while in the taxi rank:	☐ Sweating<br>☐ Heat cramps<br>☐ Headaches<br>☐ A faster heartbeat<br>☐ Nausea / vomiting<br>☐ Heat rash / pimples or blisters on skin<br>☐ Heat exhaustion / feeling tired or weak<br>☐ Dizziness or confusion<br>☐ Difficulties breathing<br>☐ Fainting<br>☐ Irritability<br>☐ None of the above<br>☐ Other - open-ended
What do you think can be done to make taxi ranks cooler and more comfortable when it is hot?	__________________________
Telephone number	__________________________
