# Supplementary material for: Heat in the transport sector: measured heat exposure and interventions to address heat-related health impacts in the minibus taxi industry in South Africa
Source: Int J Biometeorol. 2025 May 13;69(10):2475–87. doi: 10.1007/s00484-025-02935-2 (PMC12540607; doi:10.1007/s00484-025-02935-2)
Supplement: Supplementary file 2 — Supplementary file2 (PDF 39.2 KB) [file 484_2025_2935_MOESM2_ESM.pdf]

Informed Consent_Taxi Driver_Durban

Record ID ______________________________

Dear Potential Participant,

We are researchers from the South African Medical Research Council and we are investigating the effects of heat for people who use taxis and taxi ranks.

This study is funded by the National Research Foundation and the South African Medical Research Council.

You are invited to participate in a research study conducted by the Environment & Health Unit of the South African Medical Research Council.

You were selected as a possible participant in this study because you are 18 years or older and you are using this taxi rank selected for the study.

PURPOSE OF THE STUDY The study asks people about heat in taxi ranks and in taxis, as well as about things people may do to try and cope with heat in these places. It also aims to trial some interventions at taxi ranks to reduce heat felt by people at taxi ranks. This study will help us understand how heat affects taxi users and taxi drivers on hot days, and what can be done to make them feel better on hot days.

PROCEDURES If you volunteer to participate in this study, we will ask you to do the following things:

Listen to the information sheet read to you, verbally agree to be in the study and consent to take part. Complete a 10-20 minute questionnaire by answering questions asked of you by a trained fieldworker. At the end of the study, we will come back to this taxi rank and display what happened during the study and the results we found.
POTENTIAL RISKS AND DISCOMFORTS There are no risks foreseen in this study. There are no risks or discomforts to you.

POTENTIAL BENEFITS TO PARTICIPANTS AND/OR TO SOCIETY You will be given a R150-00 voucher in appreciation for your time. The study results will be analysed, anonymously (with no personal information), and displayed in this taxi rank at the end of the study. The findings will help inform a Guide on how to climate proof taxi ranks for heat impacts.

CONFIDENTIALITY We will not request any of your personal information such as your name or address. We will ask for your telephone number if you would like to participate in another part of this study later on. Data and information will be kept confidential however confidentiality cannot be guaranteed.

Any information that is obtained in connection with this study will remain confidential. All of your data will be identified only by unique identifier codes to safeguard your data. Data will be kept on an encrypted hard drive and only the PI, Co-PIs as well as the biostatistician will have access to it. Results of the study will only be reported on aggregated, anonymous data.

PARTICIPATION AND WITHDRAWAL You can choose whether to be in this study or not. If you volunteer to be in this study, you may withdraw at any time without consequences of any kind. You may refuse to answer questions you don't want to answer and still remain in the study. The investigator may withdraw you from this research if circumstances arise which warrant doing so.

IDENTIFICATION OF INVESTIGATORS If you have any questions or concerns about the research, please feel free to

contact Dr Caradee Wright on tel. (012) 339 8543; email: cwright@mrc.ac.za.

Participants can also contact the Chairperson of the SAMRC Human Research Ethics Committee should they have queries or problems (Adri Labuschagne, tel. (021) 938 0687; e-mail: adri.labuschagne@mrc.ac.za)

RIGHTS OF RESEARCH PARTICIPANTS You may withdraw your consent at any time and discontinue participation without penalty. You are not waiving any legal claims, rights or remedies because of your participation in this research study.

CONSENT

In accordance with the provisions of the Protection of Personal Information Act 4 of 2013 (as amended), I hereby consent:

a. To my personal information (hereinafter 'data') being collected, processed, shared and stored in accordance with the research protocol as approved by the South African Medical Research Council's Human Research Ethics Committee (SAMRC HREC);

b. To my anonymised data being shared, processed and transferred by third parties and between third parties, and where relevant beyond the jurisdictional borders of South Africa;

c. To all findings and results flowing from my anonymised data being broadly shared and published on the conclusion of the research.

The information above was described to me and I am in command of this language or it was satisfactorily translated to me. I was given the opportunity to ask questions and these questions were answered to my satisfaction. I hereby consent voluntarily to participate in this study (select one):

I have read this informed consent document and the material contained in it has been explained to me verbally. All my questions have been answered, and I freely and voluntarily choose to participate.

◯ Yes, I agree to participate
◯ No, I do not wish to participate

Date

____

Participant's first name and surname:

____

Participant signature

____
