# Supplementary material for: Heat in the transport sector: measured heat exposure and interventions to address heat-related health impacts in the minibus taxi industry in South Africa
Source: Int J Biometeorol. 2025 May 13;69(10):2475–87. doi: 10.1007/s00484-025-02935-2 (PMC12540607; doi:10.1007/s00484-025-02935-2)
Supplement: Supplementary file 3 — Supplementary file3 (PDF 44.1 KB) [file 484_2025_2935_MOESM3_ESM.pdf]

Rank Observation Checklist_Durban

Record ID	__________________________
Type of Taxi Rank	◯ Low Use Rank (Taxi rank where there are predominantly low passenger volumes. are generally located in suburban areas)<br>◯ Medium Use Rank (Taxi rank where there are moderate passenger volumes. These ranks are predominantly located in suburban areas such as Shopping Centres and Community Centres)<br>◯ High Use Rank (Taxi rank servicing locations. They are predominantly located at major attractions such as City and Town CBD's, Sporting Venues, Entertainment Precincts, Hotels, Clubs and Major Office Centres. Often a high use rank is provided close to bus stop infrastructure.)
How many destinations does the rank service?	__________________________
What is the average queuing time at the rank?	__________________________
What are the pick times at the rank?	__________________________
How many taxis use the facility at any given time, given observations?	__________________________
Does the rank have a commuter waiting area?	◯ Yes<br>◯ No
Does the waiting area have shade?	◯ Yes<br>◯ No
Does the rank have custom designed shelters?	◯ Yes<br>◯ No
Are taxi rank shelters sufficient to accommodate the required number of waiting commuters?	◯ Yes<br>◯ No
Does the taxi rank have pedestrian infrastructure including links to pedestrian crossings?	◯ Yes<br>◯ No
Does the rank have seating?	◯ Yes<br>◯ No
Does the rank have rubbish bins?	◯ Yes<br>◯ No
Does the rank have queuing rails?	◯ Yes<br>◯ No

Does the taxi rank have vendors' stalls/shops?	◯ Yes<br>◯ No
Do the vendors have shelter	◯ Yes<br>◯ No
Does the rank have running water for drinking?	◯ Yes<br>◯ No
Does the rank have restrooms / toilet facilities?	◯ Yes<br>◯ No
Are entry and exit points clearly identifiable?	◯ Yes<br>◯ No
Are there any trees that provide shade?	◯ Yes<br>◯ No
How many trees can you see in the taxi rank area	______________________
How tall are the trees?	☐ Under roof height ☐ Roof height<br>☐ Above roof height
Leaves of the trees	☐ Deciduous ☐ Evergreen
Rank grounds - % Grass/Greening	______________________
Rank grounds - % bare sand	______________________
Rank grounds - % concrete	______________________
Rank grounds - % paving	______________________
Rank grounds - % tar	______________________
Is space available to provide a roofed area for queuing passengers to protect them from the weather?	◯ Yes<br>◯ No
