# Supplementary material for: Heat in the transport sector: measured heat exposure and interventions to address heat-related health impacts in the minibus taxi industry in South Africa
Source: Int J Biometeorol. 2025 May 13;69(10):2475–87. doi: 10.1007/s00484-025-02935-2 (PMC12540607; doi:10.1007/s00484-025-02935-2)
Supplement: Supplementary file 4 — Supplementary file4 (DOCX 19.9 KB) [file 484_2025_2935_MOESM4_ESM.docx]

Table S1. Results of the taxi rank observation checklist.

| **Total number of taxi ranks (N= 12)** | **Frequency** | |
| --- | --- | --- |
| **Question** | **Number (n)** | **Percentage (%)** |
| **What is the type of the taxi rank?**  Low Use Rank  Medium Use Rank  High Use Rank | 6  1  5 | 50  8  42 |
| **How many destinations does the rank service?**  1 destination  2 destinations  7 destinations | 10  1  1 | 83  8  8 |
| **What are the pick times at the rank?**  06:00-17:00  06:00-19:00  06:00-20:00  06:00-20:00 & 16:00-19:00  07:00-17:00  07:00-18:00  07:00-20:00  08:00-18:00  08:00-19:00 | 1  2  1  2  1  1  1  2  1 | 8  16  8  16  8  8  8  16  8 |
| **How many taxis use the facility at any given time, given observations?**  0-10 taxis  11-20 taxis  21-30 taxis  31-40 taxis  41-50 taxis  51-60 taxis | 1  6  1  3  0  1 | 8  50  8  25  0  8 |
| **Does the rank have a commuter waiting area?**  Yes  No | 6  6 | 50  50 |
| **Does the waiting area have shade?**  Yes  No  Missing data | 4  1  7 | 33  8  58 |
| **Does the rank have custom designed shelters?**  Yes  No | 3  9 | 25  75 |
| **Are taxi rank shelters sufficient to accommodate the required number of waiting commuters?**  Yes  No | 3  9 | 25  75 |
| **Does the taxi rank have pedestrian infrastructure including links to pedestrian crossings?**  Yes  No | 5  7 | 42  58 |
| **Does the rank have seating?**  Yes  No | 0  12 | 0  100 |
| **Does the rank have rubbish bins?**  Yes  No | 4  8 | 33  67 |
| **Does rank have queuing rails?**  Yes  No  Missing data | 4  7  1 | 33  58  8 |
| **Does the rank have vendors' stalls/shops?**  Yes  No | 6  6 | 50  50 |
| **Do the vendors' stalls/shops have shelter?**  Yes  No  Missing data | 3  3  6 | 25  25  50 |
| **Does the rank have running water for drinking?**  Yes  No | 0  12 | 0  100 |
| **Does the rank have restrooms/toilet facilities?**  Yes  No | 5  7 | 42  58 |
| **Are entry and exit points clearly identifiable?**  Yes  No | 7  5 | 58  42 |
| **Are there any trees that provide shade?**  Yes  No | 6  1 | 6  1 |
| **How many trees can you see in the taxi rank area?**  0-5 trees  6-10 trees  11-15 trees | 9  2  1 | 75  17  8 |
| **What is the height of the trees?**  Under roof height  Roof height  Above roof height | 0  3  8 | 0  25  75 |
| **What is the leaf type of the trees?**  Deciduous leaves  Evergreen leaves | 0  10 | 0  83 |
| **What is the percentage of the grass/greening on the rank grounds?**  0-20 % Grass/greening  21-40 % Grass/greening  41-60 % Grass/greening  0-20 % Bare/sand  0-20 % Concrete  61-80 % Concrete  0-20 % Paving  21-40 % Tar  41-60 % Tar  61-80 % Tar  81-100 % Tar | 3  3  1  1  1  1  7  1  1  8  1 | 25  25  8  8  8  8  58  8  8  67  8 |
| **Is there space available to provide a roofed area for queuing passengers to protect them from the weather?**  Yes  No | 6  1 | 92  8 |
