# Supplementary material for: Heat in the transport sector: measured heat exposure and interventions to address heat-related health impacts in the minibus taxi industry in South Africa
Source: Int J Biometeorol. 2025 May 13;69(10):2475–87. doi: 10.1007/s00484-025-02935-2 (PMC12540607; doi:10.1007/s00484-025-02935-2)
Supplement: Supplementary file 5 — Supplementary file5 (PDF 480 KB) [file 484_2025_2935_MOESM5_ESM.pdf]

TOO HOT? Throw shade on heat this summer

CITY OF TSHWANE

SAMRC
advancing life

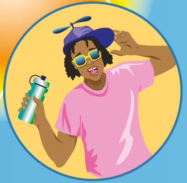

Take a water bottle with you everywhere.

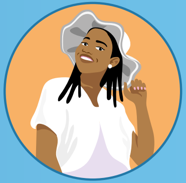

Overdressed? Wear loose light clothing.

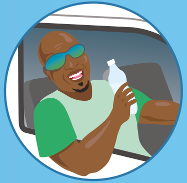

Open windows while you are travelling.

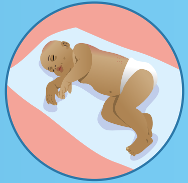

Look out for signs of illnesses heat causes.

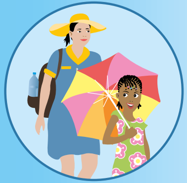

Open an umbrella or wear a hat.

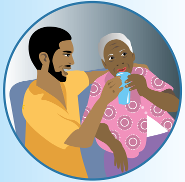

Take care of babies, our elderly and others who ask for help.
