# Supplementary material for: Heat in the transport sector: measured heat exposure and interventions to address heat-related health impacts in the minibus taxi industry in South Africa
Source: Int J Biometeorol. 2025 May 13;69(10):2475–87. doi: 10.1007/s00484-025-02935-2 (PMC12540607; doi:10.1007/s00484-025-02935-2)

SAMRC advancing life

CITY OF TSHWANE IGNITING EXCELLENCE

Throw shade on heat this summer

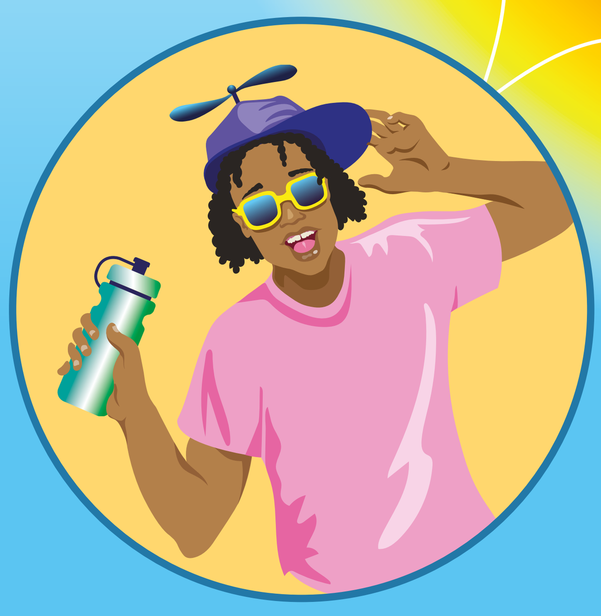

Take a water bottle with you everywhere.

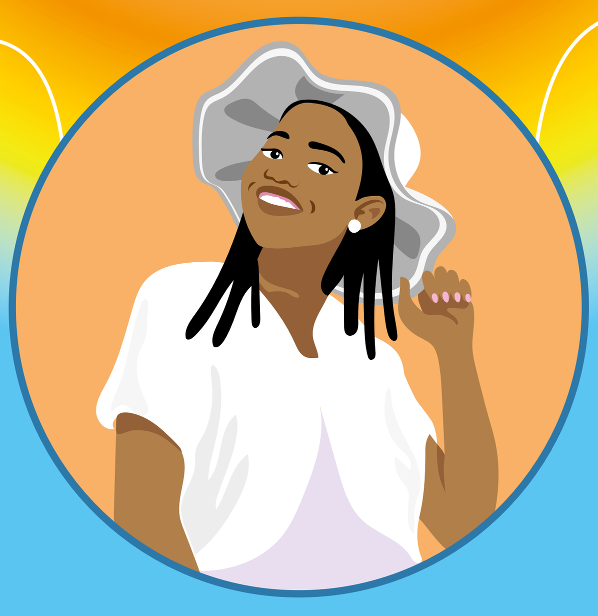

Overdressed? Wear loose light clothing.

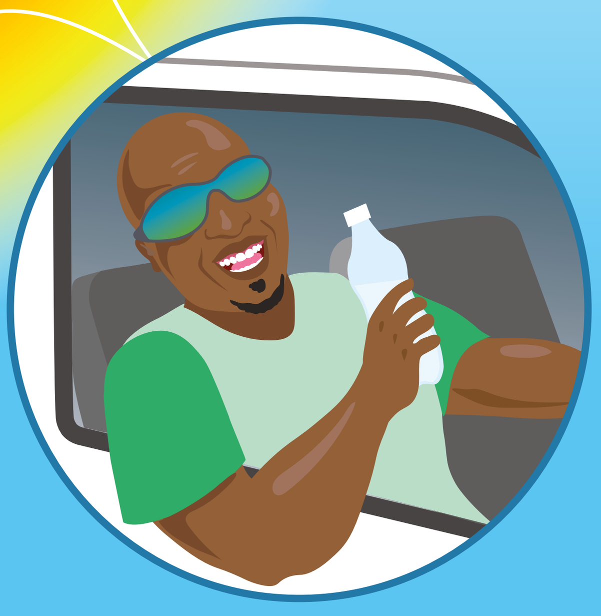

Open windows while you are travelling.

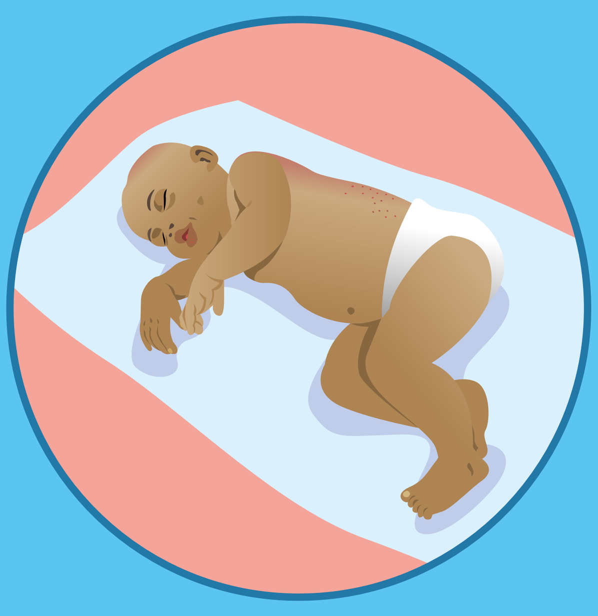

Look our for signs of illnesses heat causes.

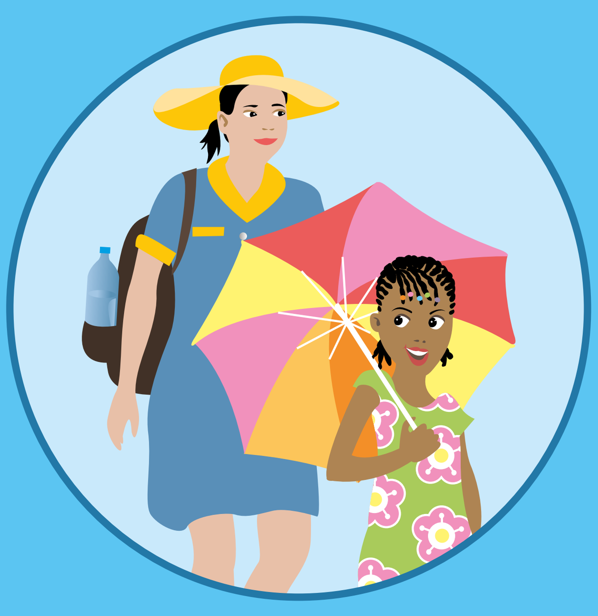

Open an umbrella or wear a hat.

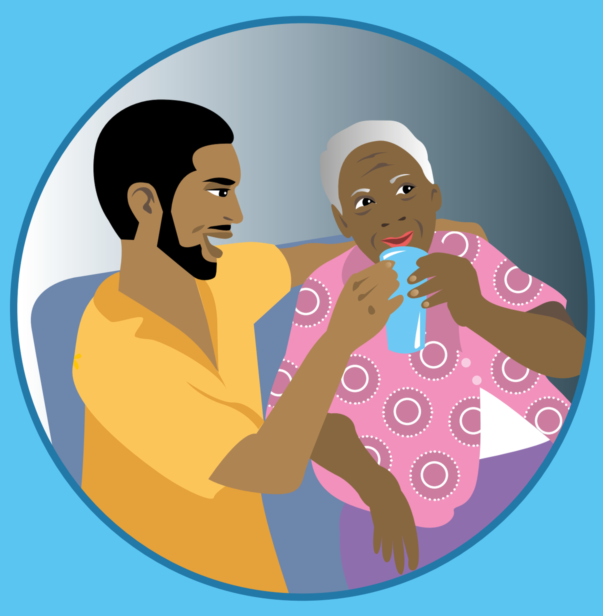

Take care of babies, our elderly and others who ask for help.

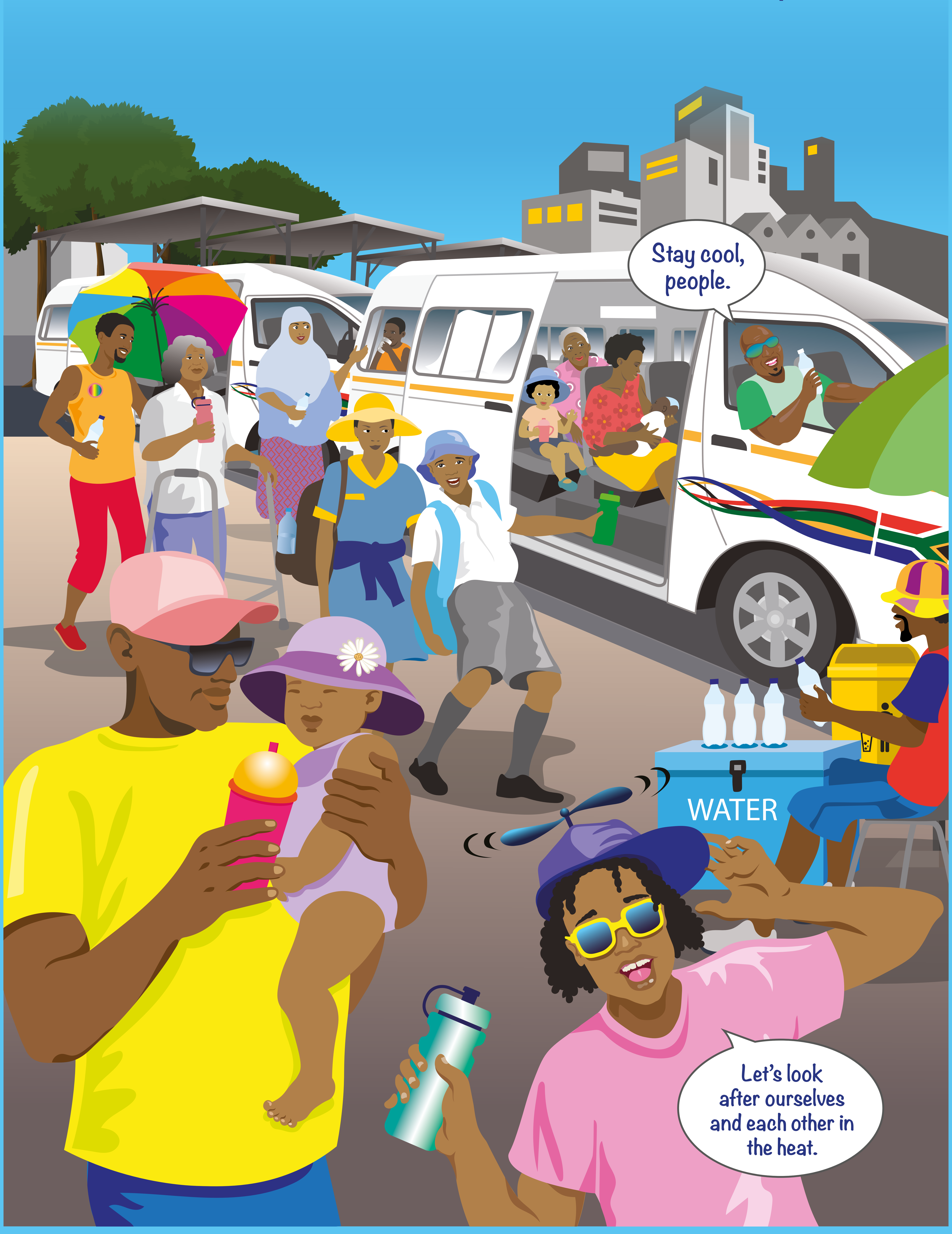

Supplement: Supplementary file 6 — Supplementary file6 (PDF 0.99 MB) [file 484_2025_2935_MOESM6_ESM.pdf]
