# Supplementary figures and images for: Heat in the transport sector: measured heat exposure and interventions to address heat-related health impacts in the minibus taxi industry in South Africa
Source: Int J Biometeorol. 2025 May 13;69(10):2475–87. doi: 10.1007/s00484-025-02935-2 (PMC12540607; doi:10.1007/s00484-025-02935-2)

90mm sticker

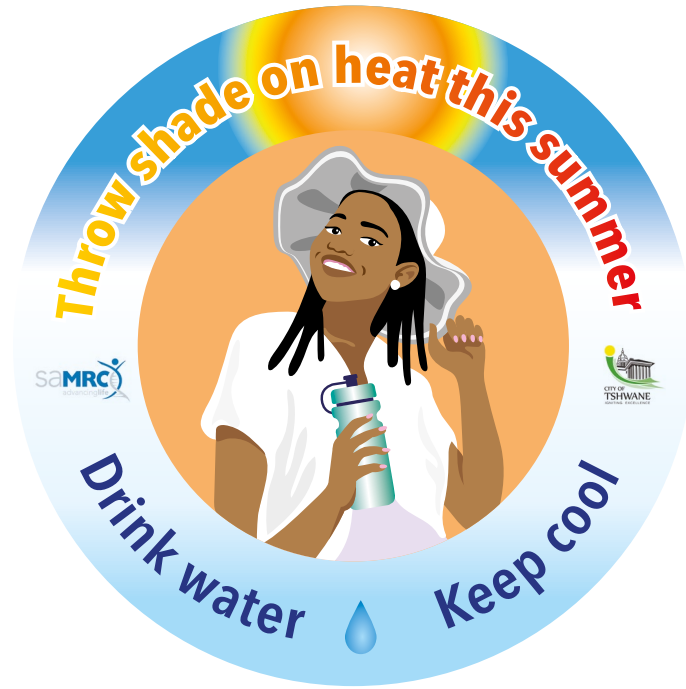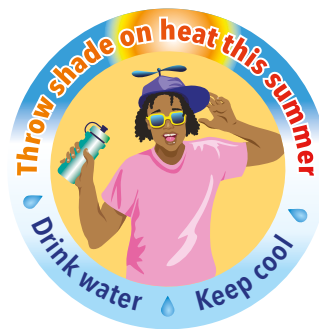

43mm badge

Supplement: Supplementary file 7 — Supplementary file7 (PDF 138 KB) [file 484_2025_2935_MOESM7_ESM.pdf]
