# Supplementary material for: Heat in the transport sector: measured heat exposure and interventions to address heat-related health impacts in the minibus taxi industry in South Africa
Source: Int J Biometeorol. 2025 May 13;69(10):2475–87. doi: 10.1007/s00484-025-02935-2 (PMC12540607; doi:10.1007/s00484-025-02935-2)
Supplement: Supplementary file 8 — Supplementary file8 (PDF 926 KB) [file 484_2025_2935_MOESM8_ESM.pdf]

CROSSWORD

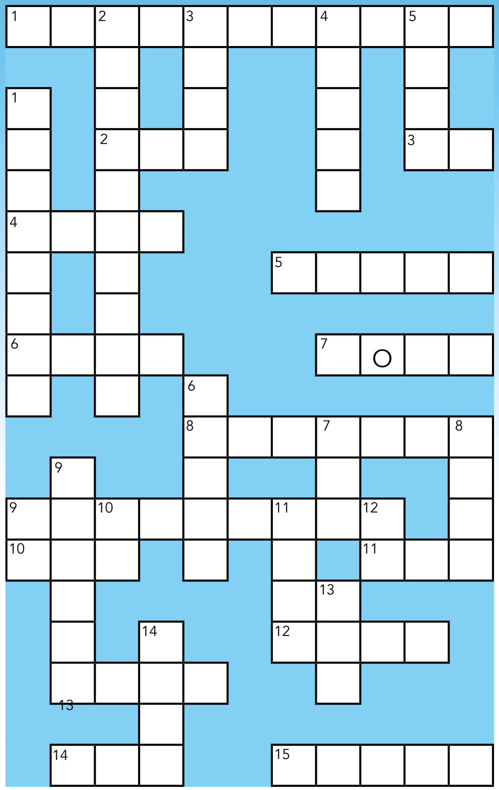

ACROSS

1. When our bodies lose water.
2. Black stuff used to put a surface on some roads.
3. The opposite of yes.
4. A stethoscope measures your heart _ _ _ _.
5. The best liquid to drink when it's hot.
6. Rhymes with bike.
7. Rhymes with pool.
8. Open them when you travel in a taxi.
9. You can get these when you have a heat illness.
10. What you make when you draw and paint.
11. You do this when you need something.
12. Sometimes you give, and sometimes you do this.
13. Another way of saying yes.
14. The opposite of night.
15. The best place to be when it is hot.

DOWN

1. Good for protection from both rain and sun.
2. A very serious heat illness.
3. Open this when the taxi is waiting for more people.
4. These help get us from one place to the next.
5. Opposite of close.
6. Hot weather makes our bodies do this.
7. A person can _ _ _ from heatstroke.
8. Heat and heat waves can make you _ _ _ _.
9. When it's hot take it off.
10. You arrive _ _ your destination.
11. This can make you ill.
12. A short way of writing South Africa.
13. When it's hot, put this on.
14. A very young, small person.

Put a cross through the drinks and food that cause more dehydration

water | alcoholic drinks
rooibos tea | sugary fizzy drinks
smoothies | fruit juice
vegetables | salty food
watermelon | sugar
fatty food | coconut water
milk | cucumber
salted popcorn | red meat
ice lollies | 'energy' drinks

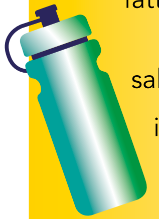

Answers: alcoholic drinks, sugary fizzy drinks, fruit juice, unless you mix half water and half juice, salty food, sugar, fatty foods, salted popcorn, red meat, 'energy' drinks.

Throw shade on heat this summer

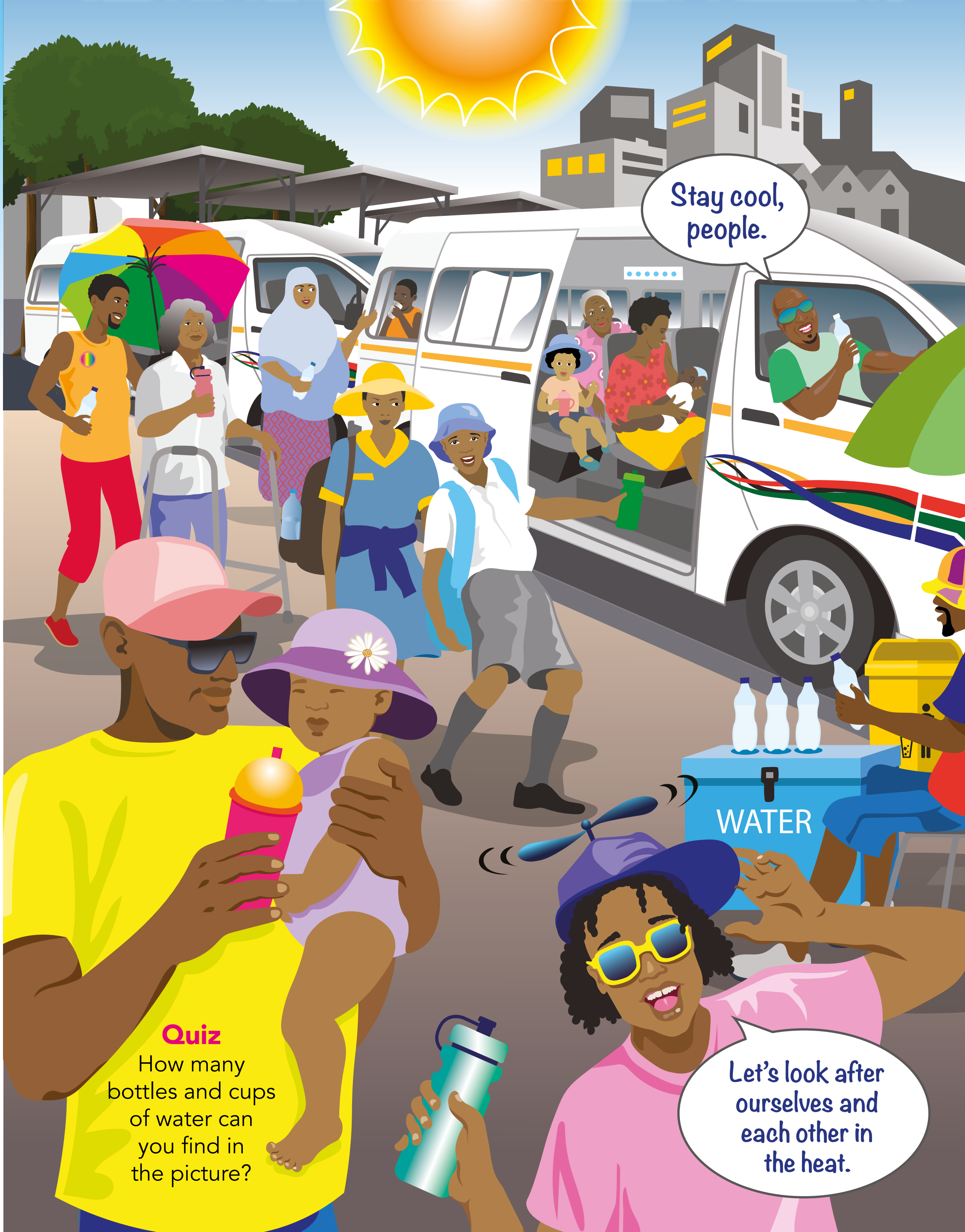

Quiz
How many bottles and cups of water can you find in the picture?

samrc advancing life
CITY OF TSHWANE IGNITING EXCELLENCE

We can get ill in hot weather

When it is very hot, your body struggles to stay at a safe temperature. Your body loses a lot of fluid when you sweat.

You need to drink lots of water and stay in the shade. Otherwise your body becomes dehydrated and can't work properly.

High temperatures are even worse for people who are pregnant, some people with disabilities, babies, and older people.

Signs of dehydration:

- dry skin, lips and mouth
- being tired a lot
- not being able to think clearly.

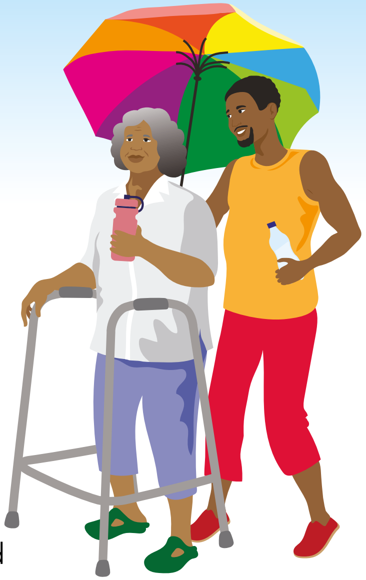

Why are older people more at risk?

Older people:

- cannot control body heat as well as before.
- often have other illnesses, such as high blood pressure, heart disease and diabetes.
- may move very quickly from being dehydrated to having heat stroke, which is very dangerous.

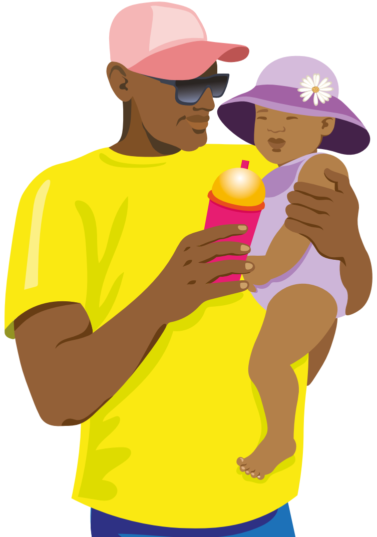

How can we prevent babies and children from getting heat illnesses?

- Do not wrap babies in blankets because this will make them sweat a lot.
- Put a cloth up at the window to give them shade.
- Babies and children must wear only loose-fitting, light clothes and a wide-brimmed hat that does not make them sweat.
- Offer babies and children cool water to drink often.

TOO HOT?

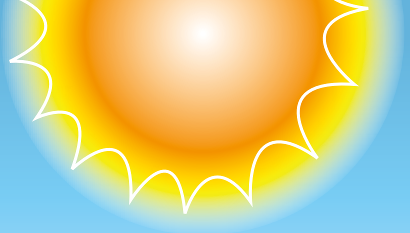

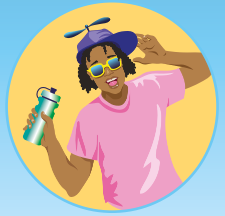

T — **Take a water bottle with you everywhere**

Hot? Drink water. Your temperature will drop.

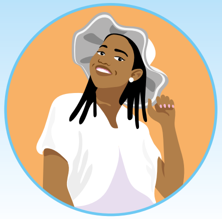

O — **Overdressed?**

Dress in loose, lightweight, light-coloured clothes. When you wear a jersey in hot weather, you will sweat more. You could dehydrate.

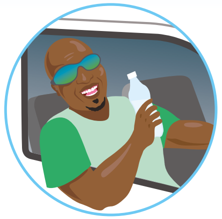

O — **Open windows while you are travelling**

Open windows and doors when the taxi is waiting. This allows a breeze to come through the taxi. It will help to bring the temperature down, and make you feel more comfortable.

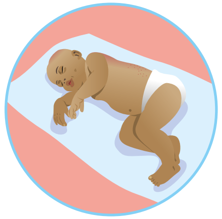

H — **Heatstroke or heat exhaustion?**

We can get very serious heat illnesses.

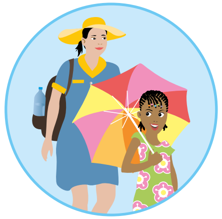

O — **Open an umbrella or wear a hat**

Umbrellas and hats give you shade. Use them while you wait in the queue.

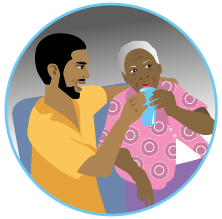

T — **Take care of babies, our elderly and others who ask for help**

Babies, people with special needs, and our elderly can very quickly get dehydrated. They can then get more serious heat illnesses. They must drink lots of water and stay in the shade.
